# A Simple and Quick Method for the Determination of Pesticides in Environmental Water by HF-LPME-GC/MS

**DOI:** 10.1155/2016/7058709

**Published:** 2016-09-28

**Authors:** Helvécio C. Menezes, Breno P. Paulo, Maria José N. Paiva, Zenilda L. Cardeal

**Affiliations:** ^1^Departamento de Química, ICEx, Universidade Federal de Minas Gerais, Avenida Antônio Carlos, 6627-31270901 Belo Horizonte, MG, Brazil; ^2^Universidade Federal de São João Del Rei, Avenida Sebastião Gonçalves Coelho 400, Chanadour, 35501-296 Divinópolis, MG, Brazil

## Abstract

This paper describes a simple and quick method for sampling and also for carrying out the preconcentration of pesticides in environmental water matrices using two-phased hollow fiber liquid phase microextraction (HF-LPME). Factors such as extraction mode, time, solvents, agitation, and salt addition were investigated in order to validate the LPME method. The following conditions were selected: 6 cm of polypropylene hollow fiber, ethyl octanoate as an acceptor phase, and extraction during 30 min under stirring at 200 rpm. The optimized method showed good linearity in the range of 0.14 to 200.00 *μ*g L^−1^; the determination coefficient (*R* ^2^) was in the range of 0.9807–0.9990. The LOD ranged from 0.04 *μ*g L^−1^ to 0.44 *μ*g L^−1^, and LOQ ranged from 0.14 *μ*g L^−1^ to 1.69 *μ*g L^−1^. The recovery ranged from 85.17% to 114.73%. The method was applied to the analyses of pesticides in three environmental water samples (a spring and few streams) collected in a rural area from the state of Minas Gerais, Brazil.

## 1. Introduction

The extensive use of pesticides harms the soil [1–4], air [5, 6], food [7–10] surface and ground waters [11–14], and quality causing serious impacts on the environment and on human health. In natural waters pesticide residues are present at very low levels and can be degraded when submitted to lower pH levels or exposed to solar radiation [15]. Furthermore the complexity of environmental matrices and large variations in physical and chemical properties of the target compounds requires the use of sensitive and selective techniques. Several analytical techniques, such as high-performance liquid chromatography (HPLC) [16, 17], gas chromatography (GC) [18, 19], micellar electrokinetic chromatography (MEKC) [20, 21], enzyme-linked immunosorbent assays (ELISA) [22–24], and gas and liquid chromatography coupled to mass spectrometry (GC/MS, LC-MS) [25, 26], have been used for analyses of pesticides in different matrices. The chromatographic techniques combine separation capabilities with sensitivity from the mass systems such as ion trap (IT), triple quadrupole (QqQ), and time of flight (TOF). However, these techniques still remain as challenges related to low detection limits, the variety of pesticides classes, and sample preparation. The analytes extraction in chromatographic analysis is critical to the method's performance since it enables the elimination of possible array interferences and the preconcentration of analytes. Traditional extraction methods such as solid phase extraction (SPE) [27, 28] and liquid-liquid extraction (LLE) are multistage consuming toxic solvents and require a long time to execute. Solid phase microextraction (SPME) [29–31] is a technique that is based on the partition between the analyte present in the matrix and the fiber coating over a small fused silica rod. This technique is solvent-free and gathers in a single step extraction and preconcentration. However problems such as low resistance, short lifetime, and high cost remain. Recently, several materials have been proposed to increase the strength and durability of SPME coatings such as carbon materials [32]. Hollow fiber liquid phase microextraction (HF-LPME) [33–37] and dispersive liquid-liquid microextraction (DLLME) [38, 39] have been used for the concentration and clean-up step of pesticides analyses in waters. HF-LPME was developed by Pedersen-Bjergaard and Rasmussen [40] and has been used by many researchers in recent years due to its low cost, which enables the rejection of the material after use, eliminating problems of cross-contamination or low reproducibility as well as its decreased consumption of organic solvents. Moreover, the process is simple and a clean-up step is not necessary and can be applied to a variety of arrays reaching high enrichment factors [41]. The technique consists of a capillary porous hydrophilic fiber, impregnated with organic solvent and its interior filled with an acceptor phase, so that it does not come into direct contact with the matrices allowing for the application of agitation during extraction [42]. HF-LPME can be used in two or three phases. With two phases the analyte is extracted from the donor through an organic solvent immiscible in water that fills the membrane pores passing to the acceptor stage, which corresponds to the same solvent [43]. With three phases the analyte is extracted from a donor phase through an organic solvent immiscible in water for an aqueous solution (acceptor phase) inside the fiber. The organic phase acts as a barrier preventing contact between the phases. Despite the extensive use of HF-LPME for extraction of pesticides in water [33, 44, 45], the reported studies using GC/MS are generally for just one pesticide class. Therefore, this study presents the development of a simple and low-cost two-phase HF-LPME methodology for multiresidue microextraction of organophosphorus, phthalimides, organochlorines, and triazoles pesticides from environmental water using GC/MS. The pesticides selected were parathion-methyl (*O,O*-dimethyl-*O-p*-nitrophenyl phosphorothioate), chlorpyrifos (*O*,*O*-diethyl* O*-3,5,6-trichloro-2-pyridyl phosphorothioate), captan (*N*-(trichloromethylthio)cyclohex-4-ene-1,2-dicarboximide), procymidone (*N*-(3,5-dichlorophenyl)-1,2-dimethylcyclopropane-1,2-dicarboximide), *α*-endosulfan (1,4,5,6,7,7-hexachloro-8,9,10-trinorborn-5-en-2,3-ylenebismethylene sulfite), prothiofos ((*RS*)-(*O*-2,4-dichlorophenyl* O*-ethyl* S*-propyl phosphorodithioate)), cyproconazole ((2*RS*,3*RS*;2*RS*,3*SR*)-2-(4-chlorophenyl)-3-cyclopropyl-1-(1*H*-1,2,4-triazol-1-yl)butan-2-ol), ethion (*O*,*O*,*O*′,*O*′-tetraethyl* S*,*S*′-methylene bis(phosphorodithioate)), triazophos (*O*,*O*-diethyl* O*-1-phenyl-1*H*-1,2,4-triazol-3-yl phosphorothioate), and phosmet (*O*,*O*-dimethyl* S*-phthalimidomethyl phosphorodithioate). The main parameters affecting the extraction efficiency were optimized using GC/MS determination. The procedure presented good accuracy and precision and low limits of quantification and detection, besides good recovery.

## 2. Materials and Methods

### 2.1. Chemical and Materials

Parathion-methyl, chlorpyrifos, captan, procymidone, *α*-endosulfan, prothiofos, cyproconazole, ethion, triazophos, and phosmet of 98% w/w purity grade were purchased from Sigma-Aldrich (St. Louis, MO, USA). The choice of pesticides was based on their use on the region of samples collection. A work solution of 20.00 mg L^−1^ was prepared by the appropriate dilution in HPLC-grade methanol, Sigma-Aldrich (St. Louis, Missouri, United States). This work solution was used for the matrix spike in different concentration levels (5.00 to 160.00 *μ*g L^−1^) to optimize the extraction conditions during the validation study. Calibration standards were prepared at 5.00, 10.00, 20.00, 40.00, 80.00, and 160.00 *μ*g L^−1^ concentrations using ultrapure water produced in a Purelab UVMK2 purifier from Elga (Marlow, Buckinghamshire‎‎, England). 1-Octanol HPLC grade was purchased from Sigma-Aldrich (St. Louis, Missouri, United States), ethyl decanoate, acetonitrile, and ethyl octanoate were purchased from J. T. Baker (Xalostoc, Edo MEX, Mexico). Hollow fiber was purchased from Underlying Performance Co. (Wuppertal, Germany).

### 2.2. Instrumentation for GC/MS

The analysis was carried out with a Shimadzu (Kyoto, Japan) GC/MS system model GC-2010/QP-2010 high-performance quadrupole. The mass spectrometer operated within the electron impact mode (EI) at 70 eV. A capillary column (30 m × 0.25 mm × 0.25 *μ*m) containing 5% diphenyl and 95% dimethylpolysiloxane HP-5MS from Agilent Technology, Inc. (Santa Clara, California, United States), was used. The oven temperature program began at 80°C and raised to 200°C at a rate of 8°C min^−1^ up to 300°C at 30°C min^−1^ and held there for 3 min. Helium (99.999%) was the carrier gas at a flow rate of 1.0 mL min^−1^. The injector was operated at 280°C in splitless mode for 3 min, followed by a 1 : 20 split ratio (RD). The ion source temperature was 200°C, and the GC/MS interface temperature was 300°C. The analysis was carried out in the selected ion monitoring (SIM) mode. The quantification was achieved using the ion fragments shown in Table 2. The collection of raw data was carried out using a LabSolution software, Shimadzu (Kyoto, Japan).

### 2.3. HF-LPME Extraction Procedure

Aqueous standards of pesticides were prepared by spiking an appropriate amount of the working standard. The extraction and desorption conditions were based on Psillakis and Kalogerakis's study [49]. LPME sampling was tested in two and three phases, study of salt addition, stirring speed, and extraction time. The extractions were carried out with propylene hollow fiber of 6.0 cm length, 600 *µ*m of internal diameter, and wall thickness of 200 *µ*m. Before the extraction, the hollow fiber was filled with 30.0 *µ*L of solvent using a microsyringe. Then, the U-shaped solvent-filled fiber was connected to two syringe needles and immersed into the vial containing 15.00 mL of aqueous donor solutions spiked with 100.0 *μ*g L^−1^ of standard pesticides solutions for the extraction under magnetic stirring. After the extraction, the acceptor phase was removed with a microsyringe and transferred to a 2.0 mL vial to the injection of 1.0 *µ*L in GC/MS. All experiments were performed in replicates (*n* = 3).

### 2.4. Samples Collection

Real samples of surface water were collected in a rural area of the state of Minas Gerais, Brazil. In this region the main crops are coffee, eucalyptus, and tomatoes. Samples of surface water were sampled 2 km downstream of these crops. The collected samples showed clear appearance and no suspended particles. The amber-glass collection bottles were previously washed with a solution of 5.0% v/v alkaline detergent under ultrasound bath for 15 minutes and rinsed with ultrapure water. Water samples collected with these bottles were carefully filled to the brim to avoid trapping air. After filling the bottles they were sealed with Teflon lined screw caps, kept in ice, and transported to the laboratory before 24 h where they were stored at 4°C until the extraction and analysis.

### 2.5. Quality Control and Quality Assurance

The quality control and the quality assurance method were carried out according to EURACHEM guidelines [50]. The limits of detection (LOD) and limits of quantification (LOQ) were calculated from mean and standard deviation of 10 blank measurements with 95% confidence. Linearity was established for all the analytes (from 0.04 to 150.0 *μ*g L^−1^). Six concentration levels were analyzed with three measurements at each concentration level. The Hartley test using Origin 8.0 from OriginLab Co. software (Northampton, MA, United States) was used to verify the instrumental response homogeneity of variances. The result of this test indicated heteroscedasticity of variances, so the linear models for the calibration curves were constructed by the least squares method weighted by the experimental variance. Intraday repeatability was calculated with four replicates. Recovery was evaluated using blank sample water spiked with 10.0 *μ*g L^−1^.

## 3. Results and Discussion

### 3.1. Optimization of HF-LPME Method

The most important factors related to the HF-LPME extraction method such as the extraction mode, solvents, agitation, salt addition, and extraction time were optimized before the validation tests. The pesticides studied were chosen based on the information of their broad use (higher quantity retailed) in the cultures of the region where the samples were collected.  Table 1 shows few relevant physicochemical properties of the selected pesticides [51].

#### 3.1.1. Extraction Mode and Solvents

The extraction mode with three phases was evaluated using acetonitrile as the acceptor phase; 1-octanol, ethyl decanoate, and ethyl octanoate were tested as the organic phase. Aqueous solutions spiked with standard pesticides were used as donor solutions.

**Figure 1:**
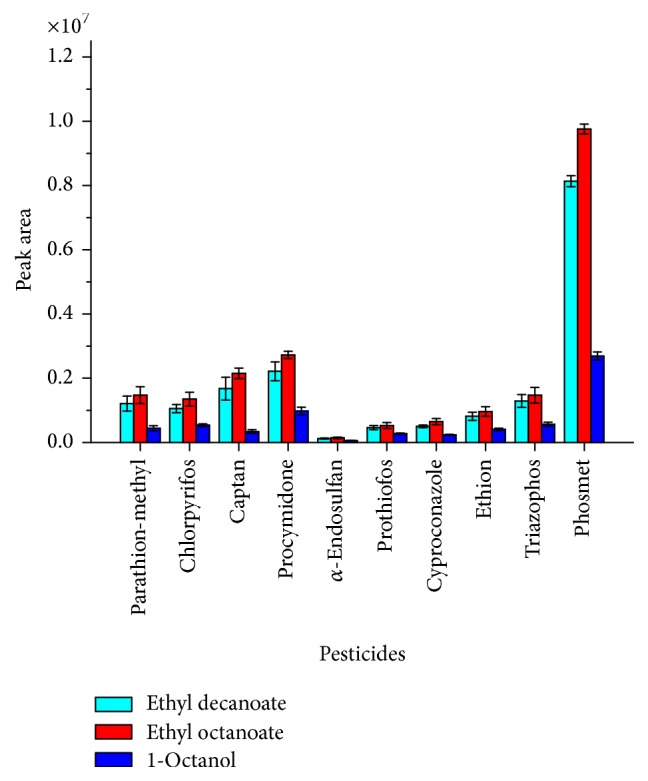
Effect of organic solvent on pesticide's extraction efficiency. Conditions of the donor phase: 15 mL of water spiked at 100 *µ*g L^−1^ of each pesticide; extraction time of 60 min; and stirring speed of 100 rpm. Error bars represented the standard deviation of the mean peak area for *n* = 3 replicates.

A significant loss of the organic phase during the three-phased extraction process significantly affected the recovery of the acceptor phase for further analysis. Subsequently, the two-phase mode was tested using 1-octanol or ethyl decanoate and ethyl octanoate as the acceptor phase and the aqueous solutions spiked with standard pesticides as donor solutions. The two-phase extraction method provided significant improvement in the recovery due to good immobilization of the acceptor phase in the fiber. Ethyl octanoate was selected as the acceptor phase due to its superior response to most pesticides as shown in Figure 1.

The results obtained with 1-octanol were relatively lower compared to the other acceptor phases studied. This can be explained by the difference in polarity between them since ethyl decanoate and ethyl octanoate are less polar than 1-octanol. Therefore, the studied analytes are best extracted in more hydrophobic solvents because all *K*_ow_ values are greater than 1. This indicates that there is greater solubility in nonpolar solvent increasing the distribution ratio between the organic acceptor solution and the donor solution [52]. The different areas observed for organochlorine pesticides in relation to organophosphorus areas are mainly due to the fact that the electron impact mode used in MS detector show extensive fragmentation. Electron impact produces relatively low signal intensity with poor sensitivity for organochlorines compounds [53].

#### 3.1.2. Agitation

**Figure 2:**
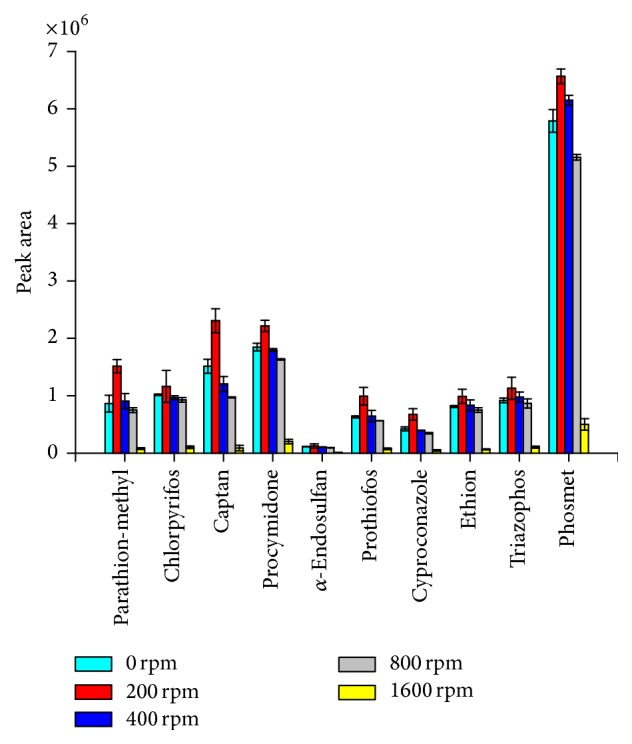
Effect of the stirring speeds on pesticide extraction efficiency. Extraction conditions of the donor phase: 15.0 mL of water spiked at 100.0 *µ*g L^−1^ of each pesticide; extraction time of 60 min; and ethyl octanoate in the acceptor phase. Error bars represented the standard deviation of the mean peak area for *n* = 3 replicates.

Agitation provides continuous exposure of the extraction surface for the aqueous sample. The fiber is depleted of the analytes due to their partitioning in the donor phase; hence, the agitation diminishes this depletion area by bringing fresh undepleted sample close to the fiber. Agitation also reduces the time required to reach thermodynamic equilibrium and induces convection in the membrane phase. To optimize sample agitation, ethyl octanoate was used as the acceptor phase, and aqueous pesticide was used as the donor phase. The stirring rates studied were 0; 200; 400; 800; and 1600 rpm using metallic stir bars of 0.5 cm. The extraction time was 60 min. The results presented in Figure 2 show that the largest areas were obtained using 200 rpm of agitation speed. It was also observed that the agitation speed higher than 200 rpm decreases extraction efficiency despite the fact that stirring promotes mass transfer between the donor and acceptor phases. The relative decreased extraction yield was due to the fact that vigorous agitation promotes the formation of air bubbles which adhere to the fiber surface [54]. The efficiency of the extraction at 0 rpm and 400 rpm are similar because in the static stage the diffusion layer close to the fiber is not renewed; this effect decreases the mass transfer to the donor layer. However, the formation of bubbles on the outer surface of the fiber starts from 400 rpm, which contribute to reducing the mass transfer of the acceptor phase.

#### 3.1.3. The Salting-Out Effect

**Figure 3:**
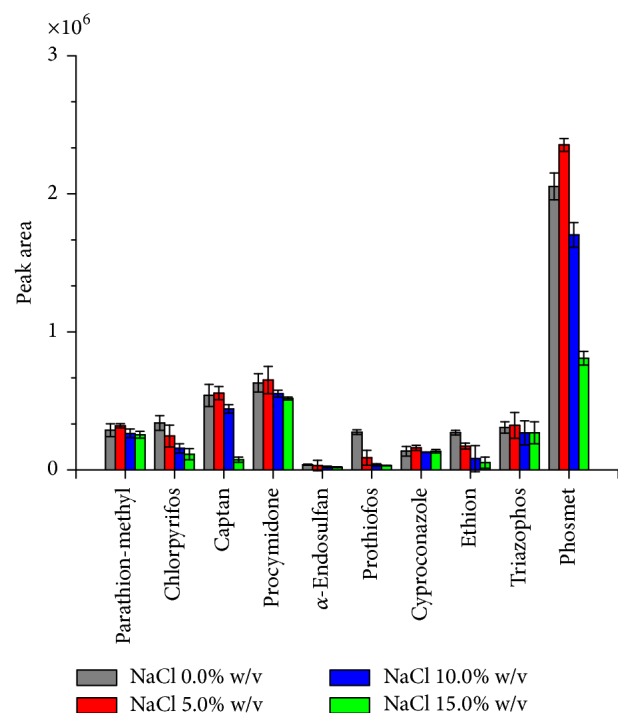
Influence of the aqueous phase ionic's strength on pesticides extraction efficiency. Extraction conditions of the donor phase: 15.0 mL of water spiked at 100.0 *µ*g L^−1^ of each pesticide with NaCl; extraction time of 60 min; stirring speed of 100 rpm; and ethyl octanoate in the acceptor phase. Error bars represented the standard deviation of the mean peak area for *n* = 3 replicates.

The effect of salt addition to the LPME extraction of the pesticides was examined in the presence of different concentrations of NaCl: 0.0; 5.0; 10.0; and 15.0% w/v. The extraction time was 60 min. The acceptor phase was ethyl octanoate with agitation at 200 rpm. The addition of salt in the aqueous samples generally improves the extraction of analytes in the organic phase. The salt increases the ionic strength decreasing the solubility of hydrophobic analytes in the donor phase; therefore, it enhances their partitioning into the acceptor phase. However, in this study the addition of salt did not present a positive influence on the extraction process of most analytes (Figure 3). Phosmet presented a large improvement in the extraction efficiency of 5.0% NaCl concentration, although at higher concentrations the peak area decreased drastically. The presence of higher concentrations of salt can change the physical properties of the extraction film reducing the diffusion rates of the analytes into the organic phase [55]. Therefore, the increase in ionic strength resulting from the addition of salt increased the salting-in effect. This effect has been observed in other studies regarding environmental water samples [35, 56].

#### 3.1.4. Extraction Time

**Figure 4:**
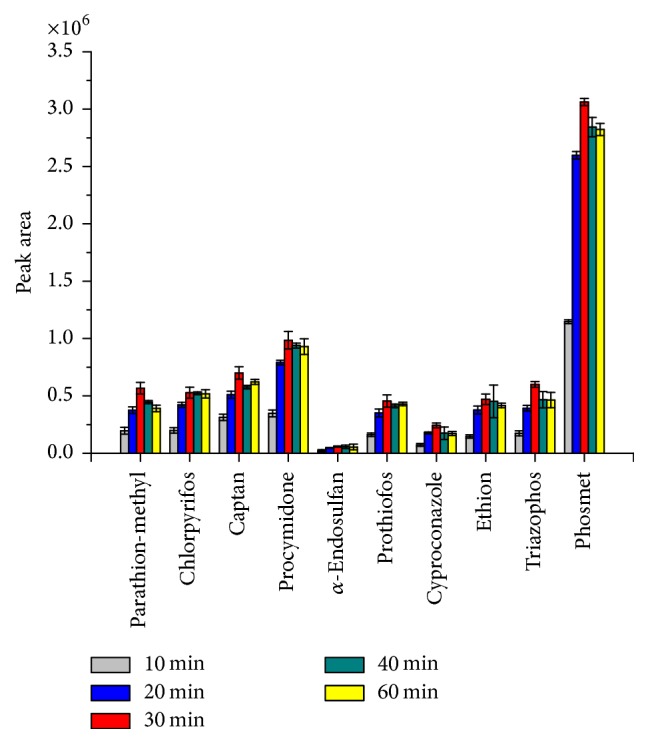
Effect exposure times on the extraction efficiency. Extraction conditions of the donor phase: 15.0 mL of water spiked at 100.0 *µ*g L^−1^ of each pesticide; stirring speed of 200 rpm; and ethyl octanoate in the acceptor phase. Error bars represented the standard deviation of the mean peak area for *n* = 3 replicates.

LPME sampling is an equilibrium process, in which analytes are partitioned between the donor phase and acceptor phase. The equilibrium time refers to the time after which the amount of extracted analyte remains constant. Extraction times of 10.0; 20.0; 30.0; 40.0; and 60.0 minutes were tested for the extraction using the best conditions of the variables previously assessed. As seen in Figure 4, the extraction efficiency reached its maximum value after 30 min for most of the pesticides evaluated. Therefore this was the extraction time selected. Periods above 30 min reduced extraction efficiency in some analytes, such as parathion-methyl, captan, phosmet, and triazophos. This reduction occurs due to the prolonging of the stirring time, which originates the formation of bubbles in the outer fiber, contributing to increased losses of the donor phase.

### 3.2. Chromatographic Evaluation

**Figure 5:**
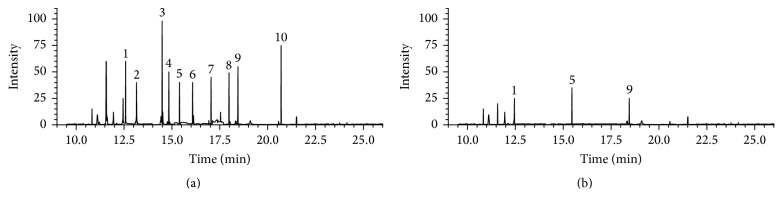
(a) Chromatogram of pesticides GC/MS analysis using HF-LPME extraction. Extraction for 30 minutes in aqueous donor solution spiked at 50.0 *μ*g L^−1^; stirring speed 200 rpm; and ethyl octanoate as acceptor phase. 1: parathion-methyl. 2: chlorpyrifos. 3: captan. 4: procymidone. 5: *α*-endosulfan. 6: prothiofos. 7: cyproconazole. 8: ethion. 9: triazophos. 10: phosmet. (b) Chromatogram of pesticides in real environmental water sample under same extraction conditions.

After evaluating the different parameters that could affect the extraction the following optimized conditions were selected for all experiments: 6 cm polypropylene hollow fiber impregnated and filled with 30 *µ*L of ethyl octanoate, immersion in a vial containing 15.0 mL of sample for 30 min under stirring at 200 rpm, and injection of 1.0 *µ*L to the acceptor phase in the GC/MS system.  Figure 5 shows chromatogram of pesticides GC/MS analysis using HF-LPME extraction in aqueous donor solution spiked at 50.0 *μ*g L^−1^ with standards (Figure 5(a)) and real environmental water sample (Figure 5(b)) under the same extraction conditions. Chromatographic separation was satisfactory for all target analytes in a short time.

Under the optimal extraction conditions ten pesticides were selected for the analysis. The parameters linearity, limits of detection (LOD), limits of quantification (LOQ), recovery, and precision were carefully investigated. The experimental results are presented in Table 2.

A linear range of 0.14 *μ*g L^−1^ to 200.00 *μ*g L^−1^ was used in the investigation. The linearity was assessed by the determination coefficient (*R* ^2^) that was in the range of 0.9807–0.9990. The LOD ranged from 0.04 *μ*g L^−1^ to 0.44 *μ*g L^−1^, and LOQ ranged from 0.14 *μ*g L^−1^ to 1.69 *μ*g L^−1^. The recovery and precision studies were performed by three replicates of real water samples spiked with concentration of 10.0 *μ*g L^−1^ of each pesticide. Recovery ranged from 85.17% to 114.73%. Intraday precision (RSD, *n* = 3) ranged from 0.90% to 15.16%. The merit parameters values of linearity and precision obtained in this study are consistent with other results reported in the analysis of pesticides in water [37, 57–61]. On the contrary, the limits of detection and quantification obtained in this study were better than those obtained in other works using HF-LPME [59, 62]. Besides, comparing other methods described in the literature (Table 3) to the multiclass pesticides analysis this HF-LPME method presents better precision and low cost.

### 3.3. Analysis of Real Environmental Samples

The application of the HF-LPME-GC/MS method for determination of pesticides of real samples was achieved through the analysis of three real samples of surface water (spring, stream 1, and stream 2) collected in a rural area of the state of Minas Gerais, Brazil.

Agriculture is the main activity in the region upstream. The concentrations determination for each pesticide in water samples are shown in Table 4.

Parathion-methyl was quantified in the sample of stream 2 and detected in spring water and stream 1. The value found for this pesticide in the samples analyzed is lower than the limit established by Brazilian legislation [63]. Triazophos was detected in all the samples analyzed. *α*-Endosulfan was detected in the spring water and stream 2. Ethion was detected in spring water and stream 1. These results show the ability of the method for the analysis of ten pesticides of different chemical classes in real samples.

## 4. Conclusions

This study describes the use of a simple and quick method for the determination of pesticides in environmental water by two-phase HF-LPME-GC/MS. The main factors related to HF-LPME extraction method such as the extraction mode, solvents, agitation, salt addition, and extraction time were investigated and optimized before the validation tests. The proposed method showed good linearity, low detection and quantification limits, high selectivity, and good repeatability for the pesticides selected. This procedure is selective, simple, fast, and low cost; it has minimal use of solvents and does not require pretreatment of samples. The results obtained from the analyses of three environmental water samples (spring and streams) have demonstrated the ability of the method to measure trace levels of pesticides. This method has the potential for automation and capacity for integrated sampling.

## Figures and Tables

**Table 1:** Physicochemical properties of the selected pesticides.

Pesticide	CAS number	Chemical class	Molecular formula	Structural formula	log⁡*K*_ow_	Water solubility (mg L^−1^)
Triazophos	24017-47-8	Organophosphorus	C_12_H_16_N_3_O_3_PS	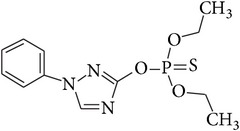	3.34	35.00
Prothiofos	34643-46-4	Organophosphorus	C_11_H_15_Cl_2_O_2_PS_2_	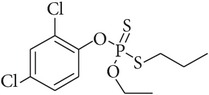	5.67	0.07
Chlorpyrifos	2921-88-2	Organophosphorus	C_9_H_11_Cl_3_NO_3_PS	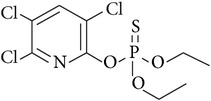	4.70	1.05
Parathion-methyl	298-00-0	Organophosphorus	C_8_H_10_NO_5_PS	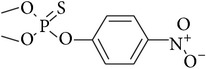	2.86	37.7
Captan	133-06-2	Dicarboximide	C_9_H_8_Cl_3_NO_2_S	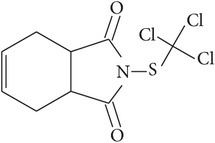	2.50	5.20
Cyproconazole	94361-06-5	Triazole	C_15_H_18_ClN_3_O	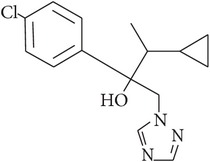	3.09	93.00
Ethion	563-12-2	Organophosphorus	C_9_H_22_O_4_P_2_S_4_	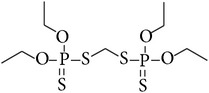	5.07	2.00
Procymidone	32809-16-8	Dicarboximide	C_13_H_11_Cl_2_NO_2_	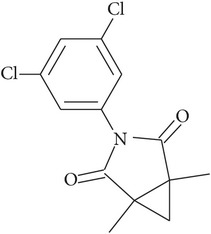	3.30	2.46
*α*-Endosulfan	115-29-7	Organochloride	C_9_H_6_Cl_6_O_3_S	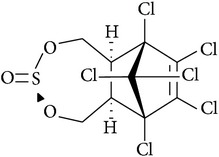	4.75	0.32
Phosmet	732-11-6	Organophosphorus	C_11_H_12_NO_4_PS_2_	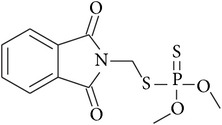	2.96	15.20

**Table 2:** Analytical performance of GC/MS method using HF-LPME for the extraction of pesticides in environmental water.

^a^Pesticide	Quantification ion (m z^−1^)	Linearity (*R* ^2^)	^b^Precision RSD (%)	^c^Recovery (%)	LOD (*µ*g L^−1^)	LOQ (*µ*g L^−1^)
Parathion-methyl	263	0.9932	2.80	110.05	0.04	0.14
Chlorpyrifos	97	0.9950	3.98	114.73	0.44	1.46
Captan	79	0.9955	14.98	100.65	0.20	0.67
Procymidone	67	0.9996	4.77	113.68	0.17	0.57
*α*-Endosulfan	195	0.9903	6.50	110.75	0.12	1.69
Prothiofos	113	0.9990	0.90	112.17	0.35	1.16
Cyproconazole	125	0.9943	9.31	85.17	0.14	0.48
Ethion	125	0.9807	2.36	113.36	0.13	0.42
Triazophos	161	0.9906	13.56	91.08	0.09	0.31
Phosmet	160	0.9874	15.16	111.10	0.23	0.76

^a^Compounds are listed in sequence of elution. ^b,c^Concentration 10 *µ*g L^−1^; *n* = 3.

**Table 3:** Comparative study for some parameters of HF-LPME-GC/MS developed method with other related methods.

Extraction method	Number of analytes	Multiclass^a^	LOD (*μ*g L^−1^)	Precision (%)	Recovery (%)	Extraction time (min)	Cost effective	Refer.
SPE	12	Yes	0.004–0.08	2.5–17.4	83–126	−	High	[28]
SPME	5	No	2.0–8.0	−	−	30	High	[31]
HF-LPME	9	No	0.002–0.012	4.2–18.4	69.4–122.7	360	Low	[35]
DLLME	18	No	0.001–0.025	5–15	75–101	0.5	Low	[39]
HF-LPME^b^	3	No	0.015–0.080	8.7–30			Low	[44]
SPME	6	Yes	0.003–0.145	4–12		60	High	[46]
SPME	16	Yes	0.015–0.13	1.9–9.6	82–114	60	High	[29]
SPME	7	Yes	0.006–0.600	2.7–23.5	60–121	80	High	[47]
SPME	16	Yes	0.02–0.30	6.3–16.9	70.2–104.6	30	High	[48]
HF-LPME	11	Yes	0.04–0.35	0.9–15.2	85.2–114.7	30	Low	This work

^a^Presence of different class of pesticides. ^b^Sample extraction of 72 h.

**Table 4:** Analyses of pesticides in real environmental water samples (spring water, stream 1, and stream 2).

Pesticide	Average (*n* = 3) concentrations (*µ*g L^−1^)			^c^Permitted value (*µ*g L^−1^)
	Spring water	Stream 1	Stream 2	
Parathion-methyl	^a^0.06	^a^0.08	0.15	35
Chlorpyrifos	^b^ND	ND	ND	^d^x
Captan	ND	ND	ND	x
Procymidone	ND	ND	ND	x
*α*-Endosulfan	^a^0.16	ND	^a^0.20	0.2
Prothiofos	ND	ND	ND	x
Cyproconazole	ND	ND	ND	x
Ethion	^a^0.30	^a^0.31	ND	x
Triazophos	^a^0.17	^a^0.14	^a^0.16	x
Phosmet	ND	ND	ND	x

^a^Below LOQ. ^b^ND: not detected. ^c^Regulation 357/2005. Ministry of Environment: pesticide levels in surface water in Brazil. ^d^x: not regulated by Brazilian legislation.

## References

[1] Fernández-Calviño D., Rodríguez-Suárez J. A., López-Periago E., Arias-Estévez M., Simal-Gándara J. (2008). Copper content of soils and river sediments in a winegrowing area, and its distribution among soil or sediment components. *Geoderma*.

[2] Pateiro-Moure M., Pérez-Novo C., Arias-Estévez M., Rial-Otero R., Simal-Gándara J. (2009). Effect of organic matter and iron oxides on quaternary herbicide sorption-desorption in vineyard-devoted soils. *Journal of Colloid and Interface Science*.

[3] Pose-Juan E., Rial-Otero R., Paradelo M., Simal-Gándara J., Arias M., López-Periago J. E. (2010). Behaviour of metalaxyl as copper oxychloride-metalaxyl commercial formulation vs. technical grade-metalaxyl in vineyards-devoted soils. *Journal of Hazardous Materials*.

[4] Pateiro-Moure M., Arias-Estévez M., Simal-Gándara J. (2013). Critical review on the environmental fate of quaternary ammonium herbicides in soils devoted to vineyards. *Environmental Science and Technology*.

[5] Kosikowska M., Biziuk M. (2010). Review of the determination of pesticide residues in ambient air. *TrAC—Trends in Analytical Chemistry*.

[6] Yusà V., Coscollà C., Millet M. (2014). New screening approach for risk assessment of pesticides in ambient air. *Atmospheric Environment*.

[7] González-Rodríguez R. M., Rial-Otero R., Cancho-Grande B., Simal-Gándara J. (2008). Occurrence of fungicide and insecticide residues in trade samples of leafy vegetables. *Food Chemistry*.

[8] González-Rodríguez R. M., Rial-Otero R., Cancho-Grande B., Gonzalez-Barreiro C., Simal-Gándara J. (2011). A review on the fate of pesticides during the processes within the food-production Chain. *Critical Reviews in Food Science and Nutrition*.

[9] López-Fernández O., Rial-Otero R., González-Barreiro C., Simal-Gándara J. (2012). Surveillance of fungicidal dithiocarbamate residues in fruits and vegetables. *Food Chemistry*.

[10] Fernández-González R., Yebra-Pimentel I., Martínez-Carballo E., Regueiro J., Simal-Gándara J. (2013). Inputs of polychlorinated biphenyl residues in animal feeds. *Food Chemistry*.

[11] Schwarzenbach R. P., Egli T., Hofstetter T. B., von Gunten U., Wehrli B. (2010). Global water pollution and human health. *Annual Review of Environment and Resources*.

[12] Bermúdez-Couso A., Fernández-Calviño D., Álvarez-Enjo M. A., Simal-Gándara J., Nóvoa-Muñoz J. C., Arias-Estévez M. (2013). Pollution of surface waters by metalaxyl and nitrate from non-point sources. *Science of the Total Environment*.

[13] Pal A., He Y. L., Jekel M., Reinhard M., Gin K. Y.-H. (2014). Emerging contaminants of public health significance as water quality indicator compounds in the urban water cycle. *Environment International*.

[14] Lari S. Z., Khan N. A., Gandhi K. N., Meshram T. S., Thacker N. P. (2014). Comparison of pesticide residues in surface water and ground water of agriculture intensive areas. *Journal of Environmental Health Science and Engineering*.

[15] Souza A. G., Costa L. M., Augusti R., Cardeal Z. L. (2010). Degradation of prototype pesticides submitted to conventional water treatment conditions: the influence of major parameters. *Water, Air, and Soil Pollution*.

[16] Jinno K., Muramatsu T., Saito Y., Kiso Y., Magdic S., Pawliszyn J. (1996). Analysis of pesticides in environmental water samples by solid-phase micro-extraction—high-performance liquid chromatography. *Journal of Chromatography A*.

[17] Masiá A., Moliner-Martinez Y., Muñoz-Ortuño M., Pico Y., Campíns-Falcó P. (2013). Multiresidue analysis of organic pollutants by in-tube solid phase microextraction coupled to ultra-high performance liquid chromatography-electrospray-tandem mass spectrometry. *Journal of Chromatography A*.

[18] Toledano R. M., Cortés J. M., Andini J. C., Villén J., Vázquez A. (2010). Large volume injection of water in gas chromatography–mass spectrometry using the Through Oven Transfer Adsorption Desorption interface: application to multiresidue analysis of pesticides. *Journal of Chromatography A*.

[19] Gonçalves C., Alpendurada M. F. (2004). Solid-phase micro-extraction-gas chromatography-(tandem) mass spectrometry as a tool for pesticide residue analysis in water samples at high sensitivity and selectivity with confirmation capabilities. *Journal of Chromatography A*.

[20] Carabias Martínez R., Rodríguez Gonzalo E., Muñoz Domínguez A. I., Domínguez Alvarez J., Hernández Méndez J. (1996). Determination of triazine herbicides in water by micellar electrokinetic capillary chromatography. *Journal of Chromatography A*.

[21] Soisungnoen P., Burakham R., Srijaranai S. (2012). Determination of organophosphorus pesticides using dispersive liquid-liquid microextraction combined with reversed electrode polarity stacking mode—micellar electrokinetic chromatography. *Talanta*.

[22] Qian G., Wang L., Wu Y. (2009). A monoclonal antibody-based sensitive enzyme-linked immunosorbent assay (ELISA) for the analysis of the organophosphorous pesticides chlorpyrifos-methyl in real samples. *Food Chemistry*.

[23] Giraudi G., Rosso I., Baggiani C., Giovannoli C., Vanni A., Grassi G. (1999). Development of an enzyme-linked immunosorbent assay for benalaxyl and its application to the analysis of water and wine. *Analytica Chimica Acta*.

[24] Zhang H., Wang S., Fang G. (2011). Applications and recent developments of multi-analyte simultaneous analysis by enzyme-linked immunosorbent assays. *Journal of Immunological Methods*.

[25] Reemtsma T., Alder L., Banasiak U. (2013). A multimethod for the determination of 150 pesticide metabolites in surface water and groundwater using direct injection liquid chromatography–mass spectrometry. *Journal of Chromatography A*.

[26] Masiá A., Ibáñez M., Blasco C., Sancho J. V., Picó Y., Hernández F. (2013). Combined use of liquid chromatography triple quadrupole mass spectrometry and liquid chromatography quadrupole time-of-flight mass spectrometry in systematic screening of pesticides and other contaminants in water samples. *Analytica Chimica Acta*.

[27] Vukcevic M., Kalijadis A., Radisic M. (2012). Application of carbonized hemp fibers as a new solid-phase extraction sorbent for analysis of pesticides in water samples. *Chemical Engineering Journal*.

[28] Vidal J. L. M., Espada M. C. P., Frenich A. G., Arrebola F. J. (2000). Pesticide trace analysis using solid-phase extraction and gas chromatography with electron-capture and tandem mass spectrometric detection in water samples. *Journal of Chromatography A*.

[29] Tankiewicz M., Morrison C., Biziuk M. (2013). Multi-residue method for the determination of 16 recently used pesticides from various chemical groups in aqueous samples by using DI-SPME coupled with GC–MS. *Talanta*.

[30] Dong C., Zeng Z., Yang M. (2005). Determination of organochlorine pesticides and their derivations in water after HS-SPME using polymethylphenylvinylsiloxane-coated fiber by GC-ECD. *Water Research*.

[31] Tomkins B. A., Ilgner R. H. (2002). Determination of atrazine and four organophosphorus pesticides in ground water using solid phase microextraction (SPME) followed by gas chromatography with selected-ion monitoring. *Journal of Chromatography A*.

[32] Ghaemi F., Amiri A., Yunus R. (2014). Methods for coating solid-phase microextraction fibers with carbon nanotubes. *TrAC—Trends in Analytical Chemistry*.

[33] Pinto M. I., Sontag G., Bernardino R. J., Noronha J. P. (2010). Pesticides in water and the performance of the liquid-phase microextraction based techniques. A review. *Microchemical Journal*.

[34] Sun X., Zhu F., Xi J. (2011). Hollow fiber liquid-phase microextraction as clean-up step for the determination of organophosphorus pesticides residues in fish tissue by gas chromatography coupled with mass spectrometry. *Marine Pollution Bulletin*.

[35] San Román I., Alonso M. L., Bartolomé L., Alonso R. M. (2012). Hollow fibre-based liquid-phase microextraction technique combined with gas chromatography-mass spectrometry for the determination of pyrethroid insecticides in water samples. *Talanta*.

[36] Tankiewicz M., Fenik J., Biziuk M. (2011). Solventless and solvent-minimized sample preparation techniques for determining currently used pesticides in water samples: a review. *Talanta*.

[37] Huang S.-P., Huang S.-D. (2007). Determination of organochlorine pesticides in water using solvent cooling assisted dynamic hollow-fiber-supported headspace liquid-phase microextraction. *Journal of Chromatography A*.

[38] Seebunrueng K., Santaladchaiyakit Y., Srijaranai S. (2014). Vortex-assisted low density solvent based demulsified dispersive liquid–liquid microextraction and high-performance liquid chromatography for the determination of organophosphorus pesticides in water samples. *Chemosphere*.

[39] Cortada C., Vidal L., Pastor R., Santiago N., Canals A. (2009). Determination of organochlorine pesticides in water samples by dispersive liquid-liquid microextraction coupled to gas chromatography-mass spectrometry. *Analytica Chimica Acta*.

[40] Pedersen-Bjergaard S., Rasmussen K. E. (2008). Liquid-phase microextraction with porous hollow fibers, a miniaturized and highly flexible format for liquid-liquid extraction. *Journal of Chromatography A*.

[41] Payán M. R., López M. Á. B., Fernández-Torres R., Mochón M. C., Ariza J. L. G. (2010). Application of hollow fiber-based liquid-phase microextraction (HF-LPME) for the determination of acidic pharmaceuticals in wastewaters. *Talanta*.

[42] Pedersen-Bjergaard S., Rasmussen K. E. (1999). Liquid-liquid-liquid microextraction for sample preparation of biological fluids prior to capillary electrophoresis. *Analytical Chemistry*.

[43] Psillakis E., Kalogerakis N. (2003). Developments in liquid-phase microextraction. *TrAC—Trends in Analytical Chemistry*.

[44] Berhanu T., Megersa N., Solomon T., Jönsson J. Å. (2008). A novel equilibrium extraction technique employing hollow fibre liquid phase microextraction for trace enrichment of freely dissolved organophosphorus pesticides in environmental waters. *International Journal of Environmental Analytical Chemistry*.

[45] Lee J., Lee H. K., Rasmussen K. E., Pedersen-Bjergaard S. (2008). Environmental and bioanalytical applications of hollow fiber membrane liquid-phase microextraction: a review. *Analytica Chimica Acta*.

[46] Pereira A., Silva E., Cerejeira M. J. (2014). Applicability of the new 60 *μ*m polyethylene glycol solid-phase microextraction fiber assembly for the simultaneous analysis of six pesticides in water. *Journal of Chromatographic Science*.

[47] Merib J. S., Simão V., Dias A. N., Carasek E. (2013). Simultaneous determination of trihalomethanes and organochlorine pesticides in water samples by direct immersion-headspace-solid phase microextraction. *Journal of Chromatography A*.

[48] Filho A. M., dos Santos F. N., Pereira P. A. D. P. (2010). Development, validation and application of a method based on DI-SPME and GC-MS for determination of pesticides of different chemical groups in surface and groundwater samples. *Microchemical Journal*.

[49] Psillakis E., Kalogerakis N. (2003). Hollow-fibre liquid-phase microextraction of phthalate esters from water. *Journal of Chromatography A*.

[50] EURACHEM (1998). *The Fitness for Purpose of Analytical Methods. A Laboratory Guide to Method Validation and Related Topics*.

[51] http://sitem.herts.ac.uk/aeru/iupac/index.htm.

[52] Rasmussen K. E., Pedersen-Bjergaard S. (2004). Developments in hollow fibre-based, liquid-phase microextraction. *TrAC—Trends in Analytical Chemistry*.

[53] Geng D., Jogsten I. E., Dunstan J. (2016). Gas chromatography/atmospheric pressure chemical ionization/mass spectrometry for the analysis of organochlorine pesticides and polychlorinated biphenyls in human serum. *Journal of Chromatography A*.

[54] Shen G., Lee H. K. (2002). Hollow fiber-protected liquid-phase microextraction of triazine herbicides. *Analytical Chemistry*.

[55] Han D., Row K. H. (2012). Trends in liquid-phase microextraction, and its application to environmental and biological samples. *Microchimica Acta*.

[56] Pan H.-J., Ho W.-H. (2004). Determination of fungicides in water using liquid phase microextraction and gas chromatography with electron capture detection. *Analytica Chimica Acta*.

[57] Xiong J., Hu B. (2008). Comparison of hollow fiber liquid phase microextraction and dispersive liquid-liquid microextraction for the determination of organosulfur pesticides in environmental and beverage samples by gas chromatography with flame photometric detection. *Journal of Chromatography A*.

[58] Gure A., Lara F. J., Megersa N., García-Campaña A. M., Del Olmo-Iruela M. (2013). Hollow-fiber liquid-phase microextraction combined with capillary HPLC for the selective determination of six sulfonylurea herbicides in environmental waters. *Journal of Separation Science*.

[59] Frenich A. G., Romero-González R., Vidal J. L. M., Ocaña R. M., Feria P. B. (2011). Comparison of solid phase microextraction and hollow fiber liquid phase microextraction for the determination of pesticides in aqueous samples by gas chromatography triple quadrupole tandem mass spectrometry. *Analytical and Bioanalytical Chemistry*.

[60] Capobiango H. L. V., Cardeal Z. L. (2005). A solid-phase microextraction method for the chromatographic determination of organophosphorus pesticides in fish, water, potatoes, guava and coffee. *Journal of the Brazilian Chemical Society*.

[61] Padrón M. E. T., Afonso-Olivares C., Sosa-Ferrera Z., Santana-Rodríguez J. J. (2014). Microextraction techniques coupled to liquid chromatography with mass spectrometry for the determination of organic micropollutants in environmental water samples. *Molecules*.

[62] Lambropoulou D. A., Albanis T. A. (2007). Liquid-phase micro-extraction techniques in pesticide residue analysis. *Journal of Biochemical and Biophysical Methods*.

[63] http://www.mma.gov.br/port/conama/res/res05/res35805.pdf.

